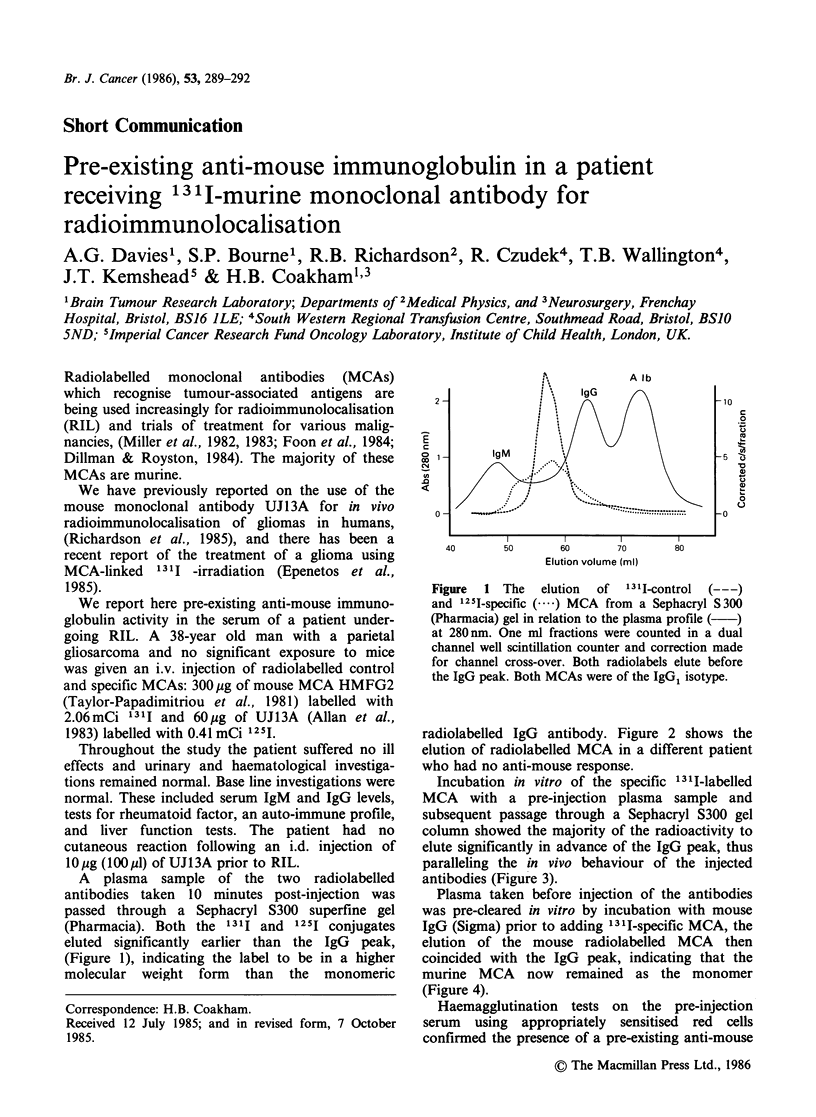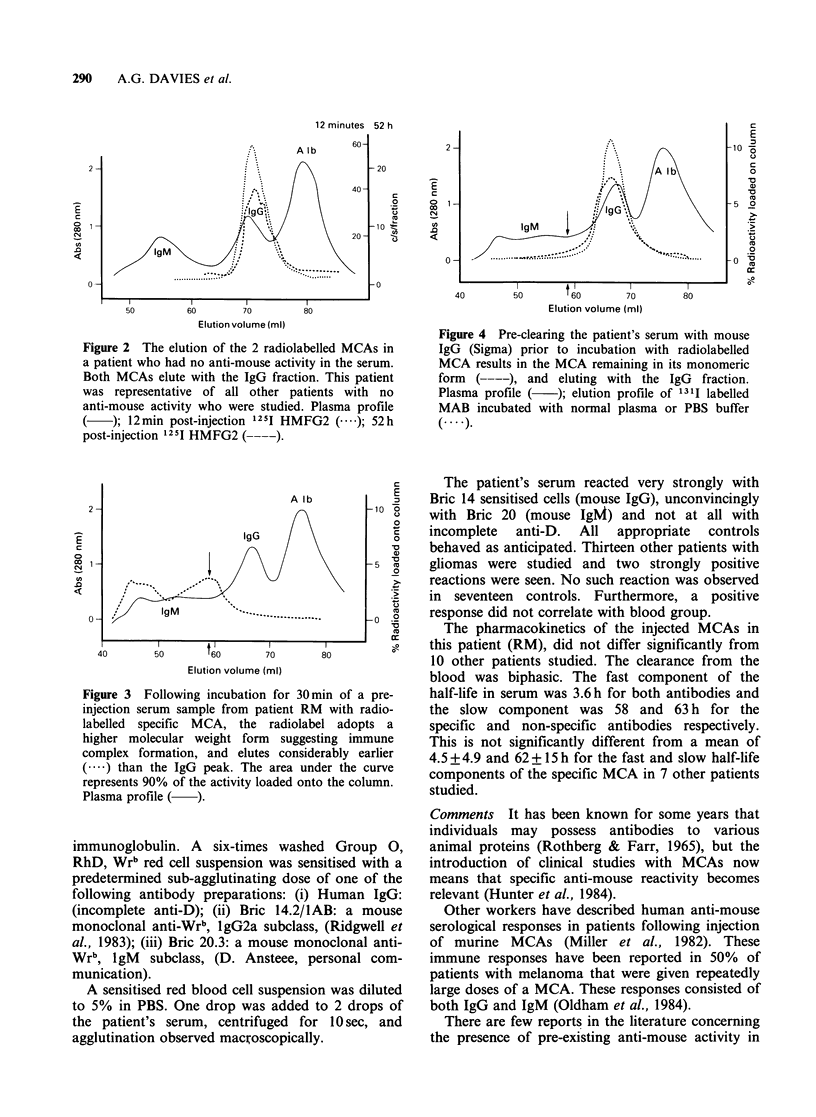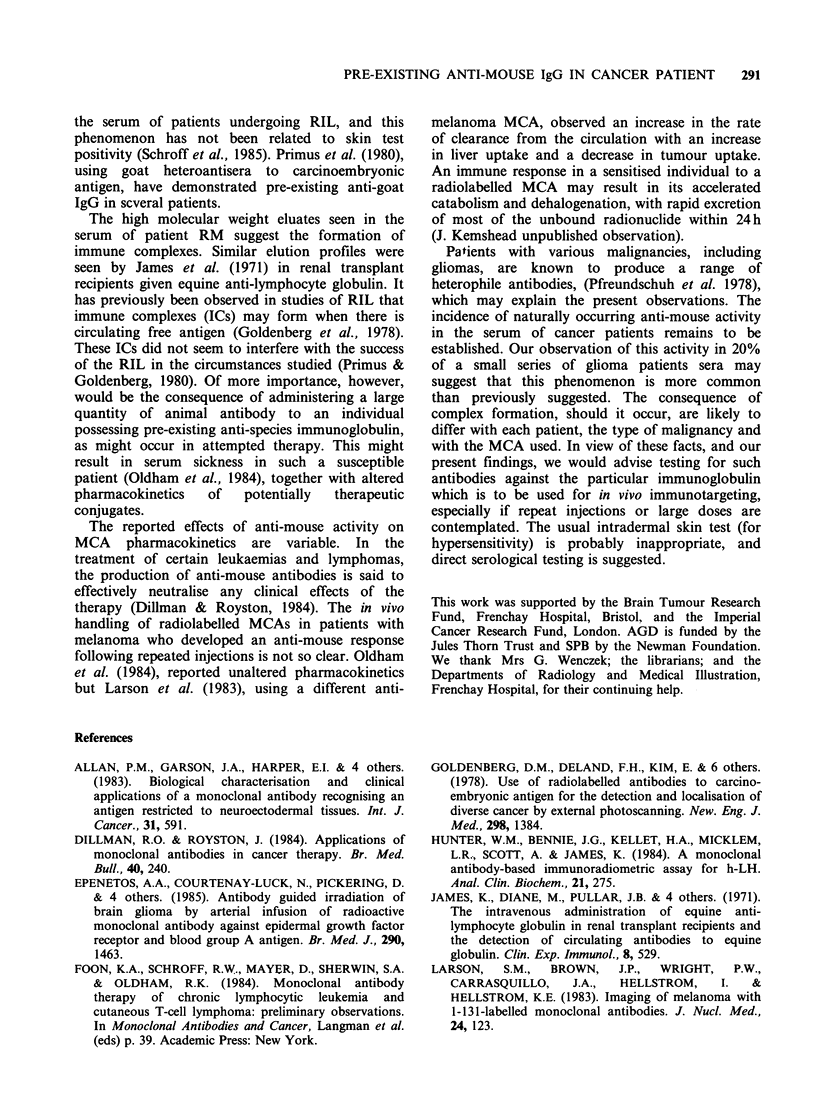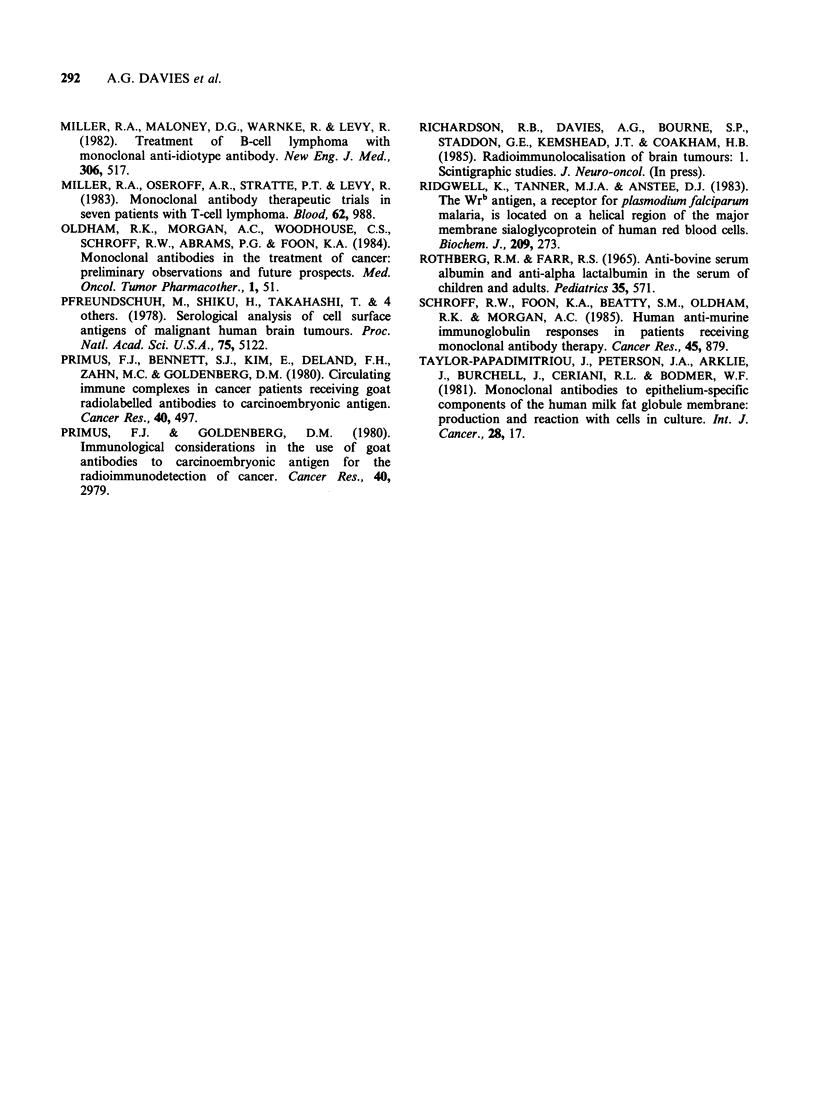# Pre-existing anti-mouse immunoglobulin in a patient receiving 131I-murine monoclonal antibody for radioimmunolocalisation.

**DOI:** 10.1038/bjc.1986.48

**Published:** 1986-02

**Authors:** A. G. Davies, S. P. Bourne, R. B. Richardson, R. Czudek, T. B. Wallington, J. T. Kemshead, H. B. Coakham


Br. J. Cancer (1986), 53, 289–292

### Short Communication

### Pre-existing anti-mouse immunoglobulin in a patient receiving $^{131}$I-murine monoclonal antibody for radioimmunolocalisation

A.G. Davies[1], S.P. Bourne[1], R.B. Richardson[2], R. Czudek[4], T.B. Wallington[4], J.T. Kemshead[5] & H.B. Coakham[1,3]

[1]*Brain Tumour Research Laboratory; Departments of* [2]*Medical Physics, and* [3]*Neurosurgery, Frenchay Hospital, Bristol, BS16 1LE;* [4]*South Western Regional Transfusion Centre, Southmead Road, Bristol, BS10 5ND;* [5]*Imperial Cancer Research Fund Oncology Laboratory, Institute of Child Health, London, UK.*

Radiolabelled monoclonal antibodies (MCAs) which recognise tumour-associated antigens are being used increasingly for radioimmunolocalisation (RIL) and trials of treatment for various malignancies, (Miller *et al.*, 1982, 1983; Foon *et al.*, 1984; Dillman & Royston, 1984). The majority of these MCAs are murine.

We have previously reported on the use of the mouse monoclonal antibody UJ13A for *in vivo* radioimmunolocalisation of gliomas in humans, (Richardson *et al.*, 1985), and there has been a recent report of the treatment of a glioma using MCA-linked $^{131}$I -irradiation (Epenetos *et al.*, 1985).

We report here pre-existing anti-mouse immunoglobulin activity in the serum of a patient undergoing RIL. A 38-year old man with a parietal gliosarcoma and no significant exposure to mice was given an i.v. injection of radiolabelled control and specific MCAs: 300 μg of mouse MCA HMFG2 (Taylor-Papadimitriou *et al.*, 1981) labelled with 2.06 mCi $^{131}$I and 60 μg of UJ13A (Allan *et al.*, 1983) labelled with 0.41 mCi $^{125}$I.

Throughout the study the patient suffered no ill effects and urinary and haematological investigations remained normal. Base line investigations were normal. These included serum IgM and IgG levels, tests for rheumatoid factor, an auto-immune profile, and liver function tests. The patient had no cutaneous reaction following an i.d. injection of 10 μg (100 μl) of UJ13A prior to RIL.

A plasma sample of the two radiolabelled antibodies taken 10 minutes post-injection was passed through a Sephacryl S300 superfine gel (Pharmacia). Both the $^{131}$I and $^{125}$I conjugates eluted significantly earlier than the IgG peak, (Figure 1), indicating the label to be in a higher molecular weight form than the monomeric radiolabelled IgG antibody. Figure 2 shows the elution of radiolabelled MCA in a different patient who had no anti-mouse response.

**Figure 1** The elution of $^{131}$I-control (---) and $^{125}$I-specific (····) MCA from a Sephacryl S300 (Pharmacia) gel in relation to the plasma profile (——) at 280 nm. One ml fractions were counted in a dual channel well scintillation counter and correction made for channel cross-over. Both radiolabels elute before the IgG peak. Both MCAs were of the $IgG_1$ isotype.

Incubation *in vitro* of the specific $^{131}$I-labelled MCA with a pre-injection plasma sample and subsequent passage through a Sephacryl S300 gel column showed the majority of the radioactivity to elute significantly in advance of the IgG peak, thus paralleling the *in vivo* behaviour of the injected antibodies (Figure 3).

Plasma taken before injection of the antibodies was pre-cleared *in vitro* by incubation with mouse IgG (Sigma) prior to adding $^{131}$I-specific MCA, the elution of the mouse radiolabelled MCA then coincided with the IgG peak, indicating that the murine MCA now remained as the monomer (Figure 4).

Haemagglutination tests on the pre-injection serum using appropriately sensitised red cells confirmed the presence of a pre-existing anti-mouse

Correspondence: H.B. Coakham.
Received 12 July 1985; and in revised form, 7 October 1985.

© The Macmillan Press Ltd., 1986

**Figure 2** The elution of the 2 radiolabelled MCAs in a patient who had no anti-mouse activity in the serum. Both MCAs elute with the IgG fraction. This patient was representative of all other patients with no anti-mouse activity who were studied. Plasma profile (——); 12 min post-injection $^{125}$I HMFG2 (····); 52 h post-injection $^{125}$I HMFG2 (– – – –).

**Figure 3** Following incubation for 30 min of a pre-injection serum sample from patient RM with radiolabelled specific MCA, the radiolabel adopts a higher molecular weight form suggesting immune complex formation, and elutes considerably earlier (····) than the IgG peak. The area under the curve represents 90% of the activity loaded onto the column. Plasma profile (——).

immunoglobulin. A six-times washed Group O, RhD, Wr$^b$ red cell suspension was sensitised with a predetermined sub-agglutinating dose of one of the following antibody preparations: (i) Human IgG: (incomplete anti-D); (ii) Bric 14.2/1AB: a mouse monoclonal anti-Wr$^b$, 1gG2a subclass, (Ridgwell *et al.*, 1983); (iii) Bric 20.3: a mouse monoclonal anti-Wr$^b$, 1gM subclass, (D. Ansteee, personal communication).

A sensitised red blood cell suspension was diluted to 5% in PBS. One drop was added to 2 drops of the patient's serum, centrifuged for 10 sec, and agglutination observed macroscopically.

**Figure 4** Pre-clearing the patient's serum with mouse IgG (Sigma) prior to incubation with radiolabelled MCA results in the MCA remaining in its monomeric form (– – – –), and eluting with the IgG fraction. Plasma profile (——); elution profile of $^{131}$I labelled MAB incubated with normal plasma or PBS buffer (····).

The patient's serum reacted very strongly with Bric 14 sensitised cells (mouse IgG), unconvincingly with Bric 20 (mouse IgM) and not at all with incomplete anti-D. All appropriate controls behaved as anticipated. Thirteen other patients with gliomas were studied and two strongly positive reactions were seen. No such reaction was observed in seventeen controls. Furthermore, a positive response did not correlate with blood group.

The pharmacokinetics of the injected MCAs in this patient (RM), did not differ significantly from 10 other patients studied. The clearance from the blood was biphasic. The fast component of the half-life in serum was 3.6 h for both antibodies and the slow component was 58 and 63 h for the specific and non-specific antibodies respectively. This is not significantly different from a mean of $4.5 \pm 4.9$ and $62 \pm 15$ h for the fast and slow half-life components of the specific MCA in 7 other patients studied.

*Comments* It has been known for some years that individuals may possess antibodies to various animal proteins (Rothberg & Farr, 1965), but the introduction of clinical studies with MCAs now means that specific anti-mouse reactivity becomes relevant (Hunter *et al.*, 1984).

Other workers have described human anti-mouse serological responses in patients following injection of murine MCAs (Miller *et al.*, 1982). These immune responses have been reported in 50% of patients with melanoma that were given repeatedly large doses of a MCA. These responses consisted of both IgG and IgM (Oldham *et al.*, 1984).

There are few reports in the literature concerning the presence of pre-existing anti-mouse activity in the serum of patients undergoing RIL, and this phenomenon has not been related to skin test positivity (Schroff *et al.*, 1985). Primus *et al.* (1980), using goat heteroantisera to carcinoembryonic antigen, have demonstrated pre-existing anti-goat IgG in scveral patients.

The high molecular weight eluates seen in the serum of patient RM suggest the formation of immune complexes. Similar elution profiles were seen by James *et al.* (1971) in renal transplant recipients given equine anti-lymphocyte globulin. It has previously been observed in studies of RIL that immune complexes (ICs) may form when there is circulating free antigen (Goldenberg *et al.*, 1978). These ICs did not seem to interfere with the success of the RIL in the circumstances studied (Primus & Goldenberg, 1980). Of more importance, however, would be the consequence of administering a large quantity of animal antibody to an individual possessing pre-existing anti-species immunoglobulin, as might occur in attempted therapy. This might result in serum sickness in such a susceptible patient (Oldham *et al.*, 1984), together with altered pharmacokinetics of potentially therapeutic conjugates.

The reported effects of anti-mouse activity on MCA pharmacokinetics are variable. In the treatment of certain leukaemias and lymphomas, the production of anti-mouse antibodies is said to effectively neutralise any clinical effects of the therapy (Dillman & Royston, 1984). The *in vivo* handling of radiolabelled MCAs in patients with melanoma who developed an anti-mouse response following repeated injections is not so clear. Oldham *et al.* (1984), reported unaltered pharmacokinetics but Larson *et al.* (1983), using a different anti-melanoma MCA, observed an increase in the rate of clearance from the circulation with an increase in liver uptake and a decrease in tumour uptake. An immune response in a sensitised individual to a radiolabelled MCA may result in its accelerated catabolism and dehalogenation, with rapid excretion of most of the unbound radionuclide within 24h (J. Kemshead unpublished observation).

Patients with various malignancies, including gliomas, are known to produce a range of heterophile antibodies, (Pfreundschuh *et al.* 1978), which may explain the present observations. The incidence of naturally occurring anti-mouse activity in the serum of cancer patients remains to be established. Our observation of this activity in 20% of a small series of glioma patients sera may suggest that this phenomenon is more common than previously suggested. The consequence of complex formation, should it occur, are likely to differ with each patient, the type of malignancy and with the MCA used. In view of these facts, and our present findings, we would advise testing for such antibodies against the particular immunoglobulin which is to be used for *in vivo* immunotargeting, especially if repeat injections or large doses are contemplated. The usual intradermal skin test (for hypersensitivity) is probably inappropriate, and direct serological testing is suggested.

This work was supported by the Brain Tumour Research Fund, Frenchay Hospital, Bristol, and the Imperial Cancer Research Fund, London. AGD is funded by the Jules Thorn Trust and SPB by the Newman Foundation. We thank Mrs G. Wenczek; the librarians; and the Departments of Radiology and Medical Illustration, Frenchay Hospital, for their continuing help.

### References

ALLAN, P.M., GARSON, J.A., HARPER, E.I. & 4 others. (1983). Biological characterisation and clinical applications of a monoclonal antibody recognising an antigen restricted to neuroectodermal tissues. *Int. J. Cancer.*, **31**, 591.

DILLMAN, R.O. & ROYSTON, J. (1984). Applications of monoclonal antibodies in cancer therapy. *Br. Med. Bull.*, **40**, 240.

EPENETOS, A.A., COURTENAY-LUCK, N., PICKERING, D. & 4 others. (1985). Antibody guided irradiation of brain glioma by arterial infusion of radioactive monoclonal antibody against epidermal growth factor receptor and blood group A antigen. *Br. Med. J.*, **290**, 1463.

FOON, K.A., SCHROFF, R.W., MAYER, D., SHERWIN, S.A. & OLDHAM, R.K. (1984). Monoclonal antibody therapy of chronic lymphocytic leukemia and cutaneous T-cell lymphoma: preliminary observations. In *Monoclonal Antibodies and Cancer*, Langman *et al.* (eds) p. 39. Academic Press: New York.

GOLDENBERG, D.M., DELAND, F.H., KIM, E. & 6 others. (1978). Use of radiolabelled antibodies to carcinoembryonic antigen for the detection and localisation of diverse cancer by external photoscanning. *New. Eng. J. Med.*, **298**, 1384.

HUNTER, W.M., BENNIE, J.G., KELLET, H.A., MICKLEM, L.R., SCOTT, A. & JAMES, K. (1984). A monoclonal antibody-based immunoradiometric assay for h-LH. *Anal. Clin. Biochem.*, **21**, 275.

JAMES, K., DIANE, M., PULLAR, J.B. & 4 others. (1971). The intravenous administration of equine anti-lymphocyte globulin in renal transplant recipients and the detection of circulating antibodies to equine globulin. *Clin. Exp. Immunol.*, **8**, 529.

LARSON, S.M., BROWN, J.P., WRIGHT, P.W., CARRASQUILLO, J.A., HELLSTROM, I. & HELLSTROM, K.E. (1983). Imaging of melanoma with 1-131-labelled monoclonal antibodies. *J. Nucl. Med.*, **24**, 123.

MILLER, R.A., MALONEY, D.G., WARNKE, R. & LEVY, R. (1982). Treatment of B-cell lymphoma with monoclonal anti-idiotype antibody. *New Eng. J. Med.*, **306**, 517.

MILLER, R.A., OSEROFF, A.R., STRATTE, P.T. & LEVY, R. (1983). Monoclonal antibody therapeutic trials in seven patients with T-cell lymphoma. *Blood*, **62**, 988.

OLDHAM, R.K., MORGAN, A.C., WOODHOUSE, C.S., SCHROFF, R.W., ABRAMS, P.G. & FOON, K.A. (1984). Monoclonal antibodies in the treatment of cancer: preliminary observations and future prospects. *Med. Oncol. Tumor Pharmacother.*, **1**, 51.

PFREUNDSCHUH, M., SHIKU, H., TAKAHASHI, T. & 4 others. (1978). Serological analysis of cell surface antigens of malignant human brain tumours. *Proc. Natl. Acad. Sci. U.S.A.*, **75**, 5122.

PRIMUS, F.J., BENNETT, S.J., KIM, E., DELAND, F.H., ZAHN, M.C. & GOLDENBERG, D.M. (1980). Circulating immune complexes in cancer patients receiving goat radiolabelled antibodies to carcinoembryonic antigen. *Cancer Res.*, **40**, 497.

PRIMUS, F.J. & GOLDENBERG, D.M. (1980). Immunological considerations in the use of goat antibodies to carcinoembryonic antigen for the radioimmunodetection of cancer. *Cancer Res.*, **40**, 2979.

RICHARDSON, R.B., DAVIES, A.G., BOURNE, S.P., STADDON, G.E., KEMSHEAD, J.T. & COAKHAM, H.B. (1985). Radioimmunolocalisation of brain tumours: 1. Scintigraphic studies. *J. Neuro-oncol.* (In press).

RIDGWELL, K., TANNER, M.J.A. & ANSTEE, D.J. (1983). The $Wr^b$ antigen, a receptor for *plasmodium falciparum* malaria, is located on a helical region of the major membrane sialoglycoprotein of human red blood cells. *Biochem. J.*, **209**, 273.

ROTHBERG, R.M. & FARR, R.S. (1965). Anti-bovine serum albumin and anti-alpha lactalbumin in the serum of children and adults. *Pediatrics* **35**, 571.

SCHROFF, R.W., FOON, K.A., BEATTY, S.M., OLDHAM, R.K. & MORGAN, A.C. (1985). Human anti-murine immunoglobulin responses in patients receiving monoclonal antibody therapy. *Cancer Res.*, **45**, 879.

TAYLOR-PAPADIMITRIOU, J., PETERSON, J.A., ARKLIE, J., BURCHELL, J., CERIANI, R.L. & BODMER, W.F. (1981). Monoclonal antibodies to epithelium-specific components of the human milk fat globule membrane: production and reaction with cells in culture. *Int. J. Cancer.*, **28**, 17.